# Hanwoo cattle: origin, domestication, breeding strategies and genomic selection

**DOI:** 10.1186/2055-0391-56-2

**Published:** 2014-05-15

**Authors:** Seung-Hwan Lee, Byoung-Ho Park, Aditi Sharma, Chang-Gwon Dang, Seung-Soo Lee, Tae-Jeong Choi, Yeon-Ho Choy, Hyeong-Cheol Kim, Ki-Jun Jeon, Si-Dong Kim, Seong-Heum Yeon, Soo-Bong Park, Hee-Seol Kang

**Affiliations:** Hanwoo Experiment Station, National Institute of Animal Science, RDA, Pyeong-Chang, 232-950 Korea; Animal Genetic and Breeding Division, National Institute of Animal Science, Cheon-An, Korea

**Keywords:** Hanwoo (Korean cattle), Origin, Domestication and breeding program

## Abstract

Hanwoo (Korean cattle) is the native, taurine type of cattle breed of Korea and its history as a draft animal dates back to 5000 Years. In earlier times Hanwoo was used extensively for farming, transportation. Over the period of time, Hanwoo has changed to be meat type cattle. Full-scale production of Hanwoo as meat-type cattle has occurred since 1960s with the rapid growth of the Korean economy. Hanwoo is one of the most economically important species in Korea as it is a significant source of nutrition to the Korean people. Hanwoo beef is the most cherished food of Korea. One of the main goals of researchers is to increase the meat quality, quantity and taste of the beef. In this review we describe the origin, domestication of Hanwoo cattle and breeding program initiated from 1980’s. Moreover the advent of technological advancement had provided us a platform to perform genome wide selection on economic traits and its implementation into traditional breeding programs.

## Introduction

**Figure 1:**
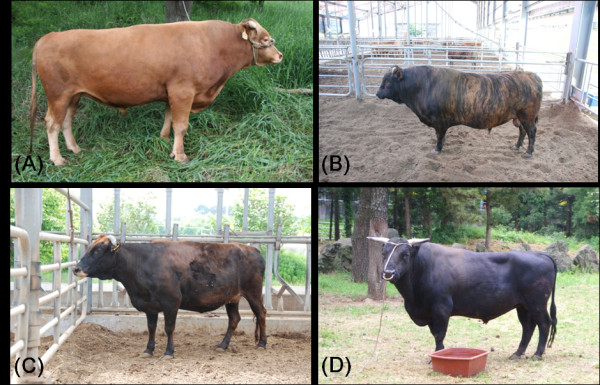
**Pictures of four Hanwoo cattle. (A)** Brown Hanwoo, **(B)** Brindle Hanwoo, **(C)** Black Hanwoo and **(D)** Jeju black Hanwoo.

Hanwoo (Korean cattle) is the native, taurine type, small sized cattle breed of Korea and its history as a draft animal dates back to 5000 Years. In earlier times Hanwoo was used extensively for farming, transportation and for religious sacrifice. Over the period of time Hanwoo cattle has evolved to be meat type cattle. Full-scale production of Hanwoo as meat-type cattle has occurred since 1960s with the rapid growth of the Korean economy. Despite its high price, beef from Hanwoo cattle is very popular among both Koreans and foreigners. Hanwoo beef is known for its marbled fat, tenderness, juiciness and characteristic flavor. There are four breeds of Hanwoo in Korea viz. brown Hanwoo, brindle Hanwoo, black Hanwoo and jeju black (Figure 1). Among all these colour variants brown is the most common one (Figure 1). National Institute of Animal Science along with the small stakeholders has taken an initiative for a better management and improvement of Hanwoo cattle. Hanwoo Experiment Station (A Research Station of National Institute of Animal Science) in Pyeongchang-gun, Gangwon-do, Korea is dedicated exclusively for research on Hanwoo cattle. Along with Hanwoo experiment station there are other institutes and universities as well which are working towards the betterment of this native cattle breed.

Korea along with Hanwoo has a market for imported beef from Australia, USA, New Zealand, Canada and Mexico as well (KMTA, [1]). When Hanwoo beef was compared with the Australian Angus, it showed characteristic compositional and quality differences that should result from genetic and environmental differences between them [2]. Hanwoo is lower in cholesterol compared to other beef. It also has a higher omega 3 fatty acid count which makes it healthier than beef from other cattle. Taste-wise, it’s very soft, juicy, and delicious. The marbling in Hanwoo steak is excellent as well and it has the right balance of meat and fat. Korean consumers decided their overall acceptability of Hanwoo beef are as follows: weights of tenderness 55%, juiciness 18%, and flavor-likeness 27% [3]. No wonder Korean consumers, despite the high price prefer Hanwoo beef to the imported one. In order to produce high quality Hanwoo beef along with specially designed breeding programs special care and feed strategies are deployed.

The major goal of researchers is to increase both the quality (Marbling, tenderness and flavor) and the quantity (Carcass weight) of the meat to benefit the Hanwoo beef industry. Therefore, the current selection index used in Korea Proven Bulls (KPN) program is based on 1) carcass weight on marbling score 2) the comparison on carcass weight. In order to support the beef industry along with the conventional selection program, the genomic selection is also being incorporated in selection and breeding programs. In the present review we discussed the origin, domestication, breeding strategies and genomic selection in Hanwoo cattle.

### Origin and Domestication

**Figure 2:**
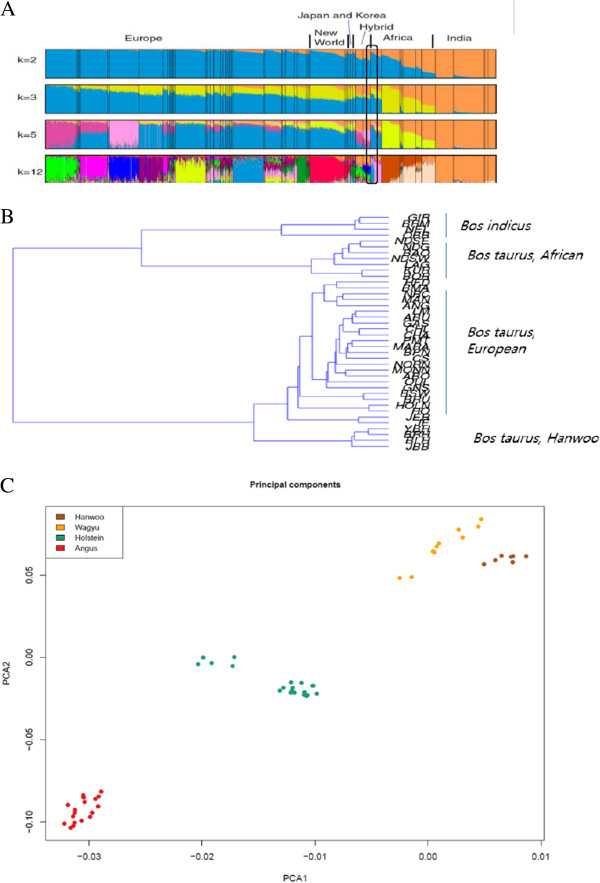
**Comparison of Hanwoo compared to other taurine cattle. (A)** Model-based population assignment for individuals based on the 50K SNP panel using STRUCTURE (K=3), YBH: Yanbian, CHB: Korean brindle, CS: Chosun, JBB: Jeju black, Ag: Angus, BS: Brown swiss, LM: Limousine, HF: Hereford, H: Holstein, BR: Brahman, HW: Hanwoo **(B)** A neighbor joining tree based on Pairwise Fst values, **(C)** Principal component analysis capturing a clear difference of north-east asian cattle.

Origin and domestication of not just Hanwoo but all north-east asian cattle is a topic of discussion among researchers. Researchers are of divided view when it comes to ancestry and domestication of Hanwoo. There are some studies on the origin and ancestry of this cattle breed. Han S. W., [4] suggested Hanwoo to be a cross between zebu and taurine cattle, which migrated to Korea from Mongolia via north china while Lee & Pollack [5] proposed Hanwoo to have originated as a hybrid from auroch and zebu cattle. Yoon et al. [6] suggested an independent domestication event for Hanwoo. Mannen et al. [7] also suggested the independent mitochondrial origin in Asian cattle. Their study included Mongolian, Korean and Japanese cattle. In a recent study McTavish et al. [8] suggested that the asian cattle (Hanwoo and Japanese Black) to be of hybrid taurine–indicine origin.We carried out cattle diversity analysis which showed a clear differentiation of asian taurine from western taurine cattle (Figure 2B-2C). Based on 50 K SNP chip data we computed a neighbor joining phylogenetic tree where we observed a clear partitioning for African taurine, European taurine, Asian taurine and Zebu cattle. The tree clearly clustered Korean cattle breeds on a separate node than the European taurine cattle breeds. This might suggest a separate domestication event for north-east asian cattle. Also, mitochondria based studies have identified a haplotype T4 that is observed in north east asian cattle breeds but not in other breeds (Mannen et al., [7]). The PCA plot also clearly separated Hanwoo and Wagyu (Japanese cattle) from Hereford and angus cattle thus supporting the inference from neighbor joining tree.

### Population genetic parameters in Hanwoo

**Figure 3:**
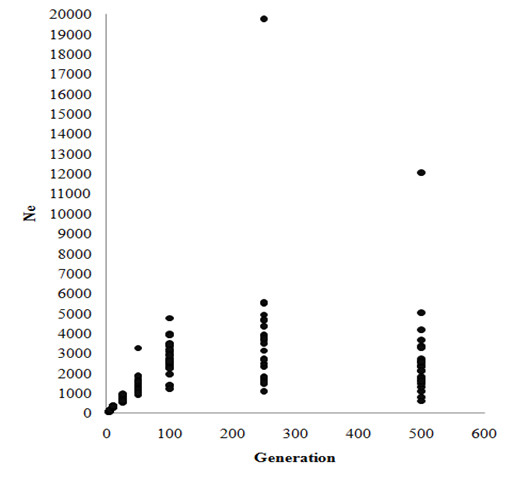
**Effective population size from chromosome of the past Hanwoo (Korean cattle) population.** Effective population size was estimated in each autosomal chromosome. The generation in the past population was calculated as 1/2c. N_e_ is plotted against the past generation up to 500 generation. (Extracted from [10]).

Linkage disequilibrium (LD) and effective population size (Ne) are important genetic parameters to understand population structure for optimal breeding program design. Lee et al., [9] performed a LD analysis and studied Ne for the entire Korean Hanwoo cattle genome, which was the first LD map and effective population size estimate ever calculated for this breed. A panel of 4,525 markers was used in the final LD analysis. The pairwise *r2* statistic of SNPs up to 50 Mb apart across the genome was estimated. A mean value of *r2* = 0.23 was observed in pairwise distances of <25 kb and dropped to 0.1 at 40 to 60 kb, which is similar to the average intermarker distance used in this study. The proportion of SNPs in useful LD (*r*^*2*^ ≥ 0.25) was 20% for the distance of 10 and 20 kb between SNPs. Analyses of past effective population size estimates based on direct estimates of recombination rates from SNP data demonstrated a decline in effective population size to *Ne* = 98.1 up to three generations ago (Figure 3). In this population genetic parameter (LD and Ne) study, the Ne in Hanwoo was larger overall than in North American Holstein cattle. In particular, the Ne of Hanwoo dramatically decreased 25 generations ago, this is when official Hanwoo breeding programs were initiated. Effect of selection pressure on Ne in the Hanwoo population was evident from this study.

### Hanwoo breeding program

Marbling score (MS), carcass weight (CWT), eye muscle area (EMA) and back fat thickness (BF) are the traits to impact Korean beef industry the most. Therefore the selection index for Korea Proven Bulls uses eye muscle area on marbling score and their comparison on CWT.

**Figure 4:**
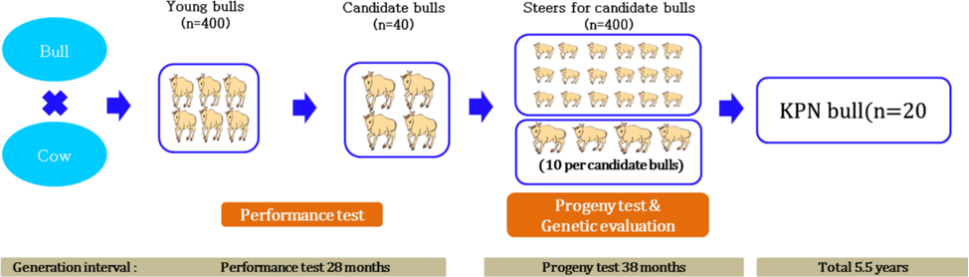
**Pictorial representation of Hanwoo performances and progeny test (HPPT) program.**

The first genetic breeding program called “Hanwoo-Gaeryang-Danji (HGD)” was initiated by Ministry of Agriculture and Forestry (MAF) in 1979. However, due to lack of awareness in the farmers to record the data and scarcity of the trained emulators, the program didn’t meet its objective. In 1999, a new program called “Hanwoo-Gaeryang-Nongga (HGN)” was introduced. Whereas HGD focused on a particular province HGN concentrated on individual farms. The cows from HGN program are used as major breeding stock for “Hanwoo Performance and Progeny Test (HPPT)” program (MAF, [11]). Prior to the HPPT program, bulls to be used for artificial insemination were selected solely on the basis of phenotypes. It was in 1987 that the first proven bull was produced using HPPT program. As shown in Figure 4, the HPPT program was a two stage selection program. The first stage was a performance test for the young bulls and the second stage was progeny test of selected young bulls. Young bull calves of current proven bulls were harvested at the age of 6 months from HGN belonging to HGD based on their phenotypic values and underwent a performance test up to age of 12 months. At this stage young bulls were selected based on a combination of their breeding values of weight at 12 months and average daily gain estimated from its performance test records. In the progeny test, cows from the HGN program were inseminated with the semen from young bulls and the male calves were harvested from farms and raised for the station progeny test. These steers were raised in a group until slaughter at 24 months. Carcass data comprised EMA, BF, MAR and CWT. Based on the progeny test results 20 bulls are selected for the AI program. Selection is based on a selection index which used weighted breeding values for BF, MAR and EMA.

Park et al. [12] carried out a study to determine the trend of improvement as well as the estimation of genetic parameters of the traits being used for seedstock selection based on the data collected from the past. In his study he concluded that the performance and progeny test based selection system of Korea is good enough to accommodate circumstances where fewer sires are used on many more cows. Although progeny tests take longer and cost more, they seem to be appropriate under the circumstances of the domestic market with its higher requirement for better meat quality. The study suggested accumulative data collection, genetic evaluation model development, revision of selection indices, as well as cooperation among farms, associations, National Agricultural Cooperative Federation, universities, research institutes and government agencies must be applied to the Hanwoo selection program. According to the Park’s study, the current progeny tested evaluation runs every 6 months to select KPN in Korea. The breeding program has achieved a substantial genetic improvement for CWT and EMA. However there has been just a slightly negative genetic response for MS with an -0.036 of genetic response per year [12].

**Figure 5:**
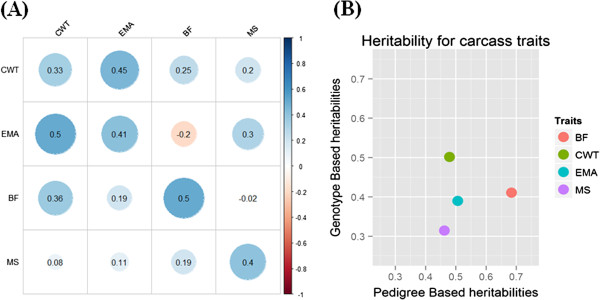
**Heritability and genetic correlation for carcass trait in Hanwoo. Data are taken from a report for Hanwoo genetic improvement meeting (2013). (A)** Along the diagonal (From upper left) are heritability values, upper triangle shows variance for genetic correlation and lower triangle shows residual variance. **(B)** Estimate of heritability from a pedigree and genotype for carcass traits in Hanwoo.

The heritability and genetic correlation are important genetic parameters in breeding program. In Hanwoo population, heritability and genetic correlation were estimated using carcass data from National Hanwoo progeny tested population (Figure 5A). A negative genetic correlation was observed between BF and EMA (-0.2) and MS and BF (-0.02) while positive correlation was observed in all other traits. A high positive correlation was observed between EMA and CWT (0.45) indicating that a greater EMA is associated with a higher production of CWT. The value of correlation between EMA and CWT in our study is more than that reported by and Koch et al. [13] and less as reported by Baik et al. [14] and Cundiff et al. [15]. High estimated heritabilities for BF (0.5), EMA (0.41), MS (0.4) and moderate for CWT (0.33) were observed. This indicates that in Hanwoo cattle a considerable genetic variability still exits which may be used for the improvement of carcass traits. Estimation of heritability, from genome-wide SNPs has recently attracted interest and offers several advantages over traditional pedigree-based methods. A comparison of pedigree and genotype based heritability for carcass traits was thus drawn (Figure 5B). The amount of variation that could be accounted for by SNP genotypes was concordant with pedigree-based heritabilities and varied from very low for CWT to medium-high for BF.

We know conventional breeding methods rely on physical characteristics or phenotypes to calculate the breeding values (BV) of animals. The traditional methods are often not so accurate, inefficient and time consuming as most traits like meat quality are hard to measure and evident only when animal reaches maturity leading to a delay in verifying breeding results. With the advent of genome wide SNP panels we can overcome the drawbacks of the conventional breeding methods. Genome wide SNP panels will allow us to accurately and cost & time effectively determine the genomic estimated breeding values (GEBV) even in young animals and thus select animals at young age.

### Genetic architecture for carcass trait in Hanwoo

Korean beef industry lay emphasis on meat yield and quality traits like carcass weight, marbling, intramuscular fat, eye muscle area. To identify the significant associations of SNPs with these traits can directly affect the selection of animals for a breeding program.

**Figure 6:**
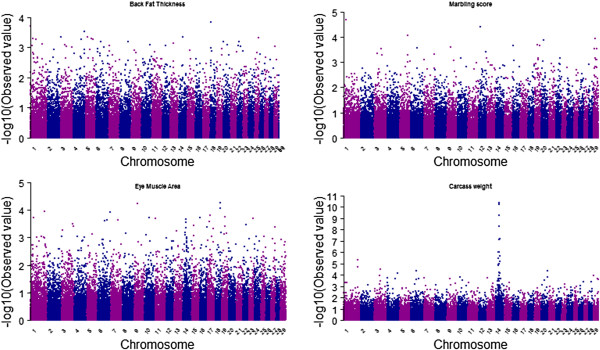
**Association of SNPs with the carcass traits in the Hanwoo shown as a Manhattan plot.**

Genome-wide association study (GWAS) is a process for inspection and screening of detectable common genetic variants (single-nucleotide polymorphisms) in individuals to identify the variant(s) associated with the trait under study. There are several GWAS studies that are successfully conducted in livestock species. A recent study on Hanwoo cattle conducted by lee et al. (2013) identified 6 highly significant SNPs on chromosome 14 to be associated with carcass weight in Hanwoo cattle (Figure 6). This genome-wide association study was conducted to identify major loci that are significantly associated with carcass weight, and their effects, so as to provide an insight into the genetic architecture of carcass weight in Hanwoo. This genome-wide association study identified a major chromosome region ranging from 23 Mb to 25 Mb on chromosome 14 as being associated with carcass weight in Hanwoo. The most significant SNP detected was *BTB-01280026* (*P = 4.02 × 10*^*-11*^), located in the 25 Mb region on Bos taurus autosome 14 (BTA14). The most significant SNPs accounted for 6.73% to 10.55% of additive genetic variance, which is quite a large proportion of the total additive genetic variance. The most significant SNP (*BTB-01280026; P = 4.02 × 10*^*-11*^) had 16.96 kg of allele substitution effect, and the second most significant SNP (*Hapmap27934-BTC-065223; P = 4.04 × 10*^*-11*^) had 18.06 kg of effect on carcass weight, which correspond to 44% and 47%, respectively, of the phenotypic standard deviation for carcass weight in Hanwoo cattle. Our results demonstrated that carcass weight was affected by a major Quantitative Trait Locus (QTL) with a large effect and by many SNPs with small effects that are normally distributed. As evident from manhattan plots of GWAS for four carcass traits shown in Figure 6 a highly significant association was observed for carcass weight on BTA14 while for other three traits there was no such association observed which shows the effect of many genes on a trait across all chromosomes.

**Figure 7:**
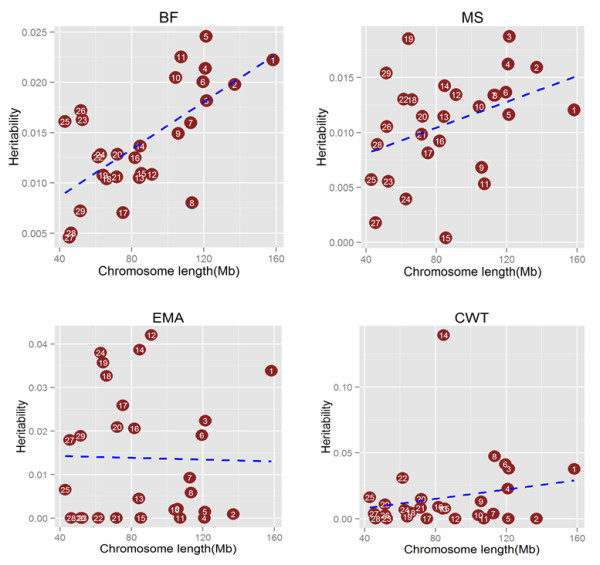
**Proportion of variance attibuted to each chromosome averaged across four carcass traits against chromosome length.**

In order to determine if polygenic inheritance is a general phenomenon for carcass traits in Hanwoo population, we directly estimated the proportion of phenotypic variance explained by the common SNPs on each chromosomes. Proportion of variance attibuted to each chromosome averaged across four carcass traits against chromosome length (Figure 7) was calculated using genotype relationship matrix (GRM) on each chromosomes which supported the results shown in manhattan plots of GWAS. While for all other traits we can see most of the chromosomes having marginal effect but for CWT where Chromosome 14 is clearly showing a high effect on the trait. By calculating the proportion of the genome represented by each chromosome (not including the length of sex chromosomes), we tested for a correlation between the variance explained by each chromosome relative to its size. It was observed that several of the smaller chromosomes contributed less to the overall variance than several of the larger chromosomes, however trend was not significant.

Also there are reports on GWAS studies on other traits of economical importance such as MS, IMF, EMA and sensory panel. Yi et al. [16] carried out a GWAS study to identify QTL for growth and carcass quality traits using high-density SNP panels. The data set comprised of 61 sires, their 486 steers, and the 54,001 SNP markers on 29 bovine autosomal chromosomes. Traits to be analyzed in this study were six growth and carcass quality traits including weaning weight (WWT), 365-d yearling weight (YWT), CWT after slaughter, BF, EMA, and MS. A total of 16(0), 18(4), 20(13), 11(23), 10(13) and 19(1) SNPs were detected at the 5% chromosome (genome)-wise level for the traits, WWT, YWT, CWT, BF, EMA and MS, respectively. Among the 148 SNPs, 91 SNPs had dominance effects, suggesting that dominance inheritance mode be considered in genetic improvement for growth and caracass quality in Hanwoo. Thirty five QTL regions on 17 Bos taurus chromosomes (i.e. BTA 3, 4, 5, 6, 7, 11, 12, 13, 14, 15, 16, 17, 18, 20, 23, 26, and 28) were detected. Strong evidence for the QTL influencing CWT were detected on BTA14. Also, the QTL for WWT, YWT, BF, and EMA were detected on BTA20. The GWAS studies could thus greatly help in developing the selection strategies for breeding programs.

### Genomic strategy for breeding scheme

Genomic selection (GS) employs selection of an individual based on the genomic breeding value assessed through evaluating all the genetic markers located throughout the genome of that individual. Thus, the underlying principle of GS is to exploit the linkage disequilibrium of the QTL with one or more genetic marker(s) [17]. Molecular markers can be used to predict GBV of breeding animals by exploiting population-wide linkage disequilibrium between QTL and genetic markers spanned over the genome. The key–factor behind the success of genomic selection is to utilize the next generation sequencing approaches and the associated bioinformatics tools to identify the SNPs. Simulation studies in some domesticated species like, beef cattle, swine and chicken (Meuwissen et al., [18]) have suggested that the breeding values can be predicted with high accuracy using genetic markers alone but its validation is required especially in samples of the population different from that in which the effect of the markers was estimated. In genomic selection, the estimation of genomic breeding values is predicted to sum up all loci that are estimated based on phenotypes and genotypes in training population. This is particularly useful for traits that are very difficult to measure, such as marbling. The accuracy of these genomic predictions depends on the genetic architecture of the complex traits. For example, number of loci affecting the trait and distribution of their effects. In case of Hanwoo, MAS (Marker Associated Selection) and GS will provide a way to predict phenotype of marbling score as a preselection method that can be used in performance test [19].

### Strategies for genomic selection program in Hanwoo

Application of genomic selection in Hanwoo preselection process such as performance test and then in progeny test in the breeding program might raise the genetic gain for marbling. A comparison of accuracy of GEBV and traditional EBV is tabulated in Table 1. In order to estimate the genomic breeding value, well organized reference population (Training data set) is to be build. The two most important features of reference population are 1) to representation of the entire Hanwoo population (all the variants) and the sample size. For the same, current progeny tested steers, selected proven bulls and candidate bulls from the progeny test program will be a reasonable population. Reference population must contain minimum of 5,000 animals. A genetic gain of 30% can be observed if we have a large reference population.

**Table 1:** **Accuracy of GEBV and traditional EBV estimated by 50 K SNP chip for Hanwoo cattle**

Traits	BLUP	GBLUP	Difference
Eye muscle area (cm^2^)	0.11(0.08)	0.29(0.07)	0.18
Back fat thickness (mm)	0.11(0.08)	0.30(0.11)	0.19
Marbling score (1 ~ 9)	0.11(0.08)	0.27(0.12)	0.16

## Conclusion

Recent advances in molecular biotechnology facilitate not only detection of genes that contribute to genetic variation of quantitative traits but also incorporation of genomic information into a conventional animal breeding program. The incorporated molecular information into GEBV may achieve an improvement of EBV and selection accuracy in cattle populations. In conclusion, we suggest incorporating molecular information into the conventional breeding programs to achieve better results in comparatively shorter time. Including molecular information will be a step further to achieve higher standards of Hanwoo beef.
